# Combining complexity measures of EEG data: multiplying measures reveal previously hidden information

**DOI:** 10.12688/f1000research.6590.1

**Published:** 2015-06-01

**Authors:** Thomas Burns, Ramesh Rajan

**Affiliations:** 1 Department of Physiology, Monash University, Melbourne, 3800, Australia

**Keywords:** electroencephalograph, complexity, complexity measure, sample entropy, permutation entropy, Lemel-Ziv complexity, fractal dimension, Weiner entropy

## Abstract

Many studies have noted significant differences among human electroencephalograph (EEG) results when participants or patients are exposed to different stimuli, undertaking different tasks, or being affected by conditions such as epilepsy or Alzheimer's disease. Such studies often use only one or two measures of complexity and do not regularly justify their choice of measure beyond the fact that it has been used in previous studies. If more measures were added to such studies, however, more complete information might be found about these reported differences. Such information might be useful in confirming the existence or extent of such differences, or in understanding their physiological bases. In this study we analysed publically-available EEG data using a range of complexity measures to determine how well the measures correlated with one another. The complexity measures did not all significantly correlate, suggesting that different measures were measuring unique features of the EEG signals and thus revealing information which other measures were unable to detect. Therefore, the results from this analysis suggests that combinations of complexity measures reveal unique information which is in addition to the information captured by other measures of complexity in EEG data. For this reason, researchers using individual complexity measures for EEG data should consider using combinations of measures to more completely account for any differences they observe and to ensure the robustness of any relationships identified.

## Introduction

Electroencephalography (EEG) is a common, relatively non-invasive research and diagnostic tool. Its one-dimensional signals from localised peripheral regions on the head make it attractive for its simplistic fidelity and has allowed high clinical and basic research throughput. When it comes to interpreting EEG data, investigators have a wide range of analytical tools at their disposal ( Dauwels *et al.*, 2010; Delorme & Makeig, 2004) and in recent years have explored a number of novel relationships between measures of complexity ( Cao & Slobounov, 2011; Dauwels *et al.*, 2011; Jing *et al.*, 2014; Sitt *et al.*, 2014; Susmáková & Krakovská, 2008; Weiss *et al.*, 2011). Studies which have included complexity measures, however, do not regularly include more than one or two such measures. For example, Dauwels *et al.* (2011) include the Lempel-Ziv (LZ) complexity measure ( Lempel & Ziv, 1976) - an algorithmic-based measure - and regularity measures, but ignore potential chaotic and fractal measures. This is not to suggest that the LZ complexity measure or that regularity measures are meaningless, nor that chaotic and fractal measures are more or less important than other measures of complexity, but that all may be measuring different features. Thus, for a more complete and robust picture of any relationships found for one complexity measure in EEG data, it might be useful for investigators to include other measures in their analyses.

This study therefore aims to determine whether different measures of complexity of EEG signals correlate, and (if so) to what degree. To do this, a small battery of complexity measures were computed for publicly-available normative data and subsequently analysed for correlations. If some measures were found not to significantly correlate or correlate fully, this would suggest that these measures are detecting unique information which might otherwise have remained hidden to investigators who were computing only a single complexity measure from their data.

## Methods

One thousand, one hundred EEG recordings of 1-second duration from 13 healthy control subjects undergoing an object recognition psychophysics task were obtained from a publicly-available database created by Begleiter (1996) of the Neurodynamics Laboratory, State University of New York Health Center, Brooklyn, United States. The control subjects were selected so as to avoid disease-specific influences. While our sample size was limited by the database, prior studies which used this database reached significance (thus, independent power calculations were not performed). Detailed demographic, subject, recording, and task information can be found in the original study by Zhang *et al.* (1995). The following complexity measures were calculated in MATLAB for each recording: LZ algorithmic complexity ( Lempel & Ziv, 1976), fractal dimension estimation (FD) ( Higuchi, 1988), permutation entropy (PE) ( Bandt & Pompe, 2002), Wiener entropy (WE) ( Wiener, 1954), and spectral structure variability (SSV) ( Singh, 2011). These measures were chosen on the basis of their broad representation of different conceptions of ‘complexity’, including informational theoretic, chaotic/fractal, and computational informatic approaches; details of how these measures are calculated and what they measure are well-described by their respective original proposers ( Bandt & Pompe, 2002; Higuchi, 1988; Lempel & Ziv, 1976; Singh, 2011; Wiener, 1954) and so will not be repeated here (see Data Availability for code details). Many more measures exist than these, however as the principle aim of this paper was to determine if differences exist at all, any differences detected in this small cross-section of measures would sufficiently illustrate this. Results from the complexity measures were analysed by linear regression and significance (considered as p<0.05) for relationships between pairs of measures was calculated using Pearson product-moment correlation coefficients. For relationships which appeared to have non-linear components when viewing its scatterplot, binomial regression was attempted. Graphs and statistics were generated using MATLAB R2012a (7.14.0.739) and Microsoft Excel 2007.

## Results

Of the ten pairs of measures, eight pairs exhibited highly significant (p<0.0001) correlations while two pairs - (i) PE and FD, (ii) WE and LZ - did not significantly correlate ( Table 1 and Table 2). High degrees of spread were noted among all correlations.

**Table 1.** Pearson (r) correlation matrix for each pair of complexity measures computed for normative EEG recordings.

	FD	LZ	WE	PE	SSV
FD	-	0.5402	0.4155	-0.0255	0.6517
LZ	0.5402	-	-0.0472	-0.1273	0.5983
WE	0.4155	0.4155	-	0.3469	0.5977
PE	-0.0255	-0.1273	0.3469	-	0.1672
SSV	0.6517	0.5983	0.5977	0.1672	-

**Table 2.** Significance (p) of correlations for each pair of complexity measures computed for normative EEG recordings.

	FD	LZ	WE	PE	SSV
FD	-	<0.0001	<0.0001	0.3990	<0.0001
LZ	<0.0001	-	0.1174	<0.0001	<0.0001
WE	<0.0001	0.1174	-	<0.0001	<0.0001
PE	0.3990	<0.0001	<0.0001	-	<0.0001
SSV	<0.0001	<0.0001	<0.0001	<0.0001	-

**Figure 1.**
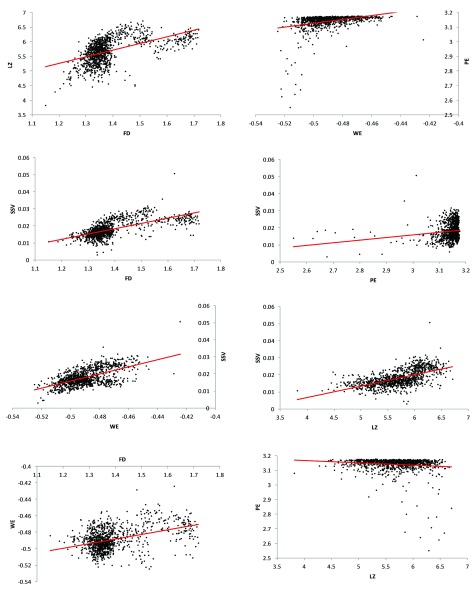
Scatter plots with linear trendlines for pairs of significantly-correlated complexity measures. Eight pairs of complexity measures of the EEG signals had a significant (p<0.0001) correlation. Although the relationships are significant, high degrees of spread are noticeable and some of the relationships may have non-linear components. *EEG = electroencephalogram; LZ = Lempel-Ziv algorithmic complexity; FD = fractal dimension estimate (Higuchi method); PE = permutation entropy; SSV = spectral structure variability; WE = Wiener entropy (also known as spectral flatness)*.

**Figure 2.**
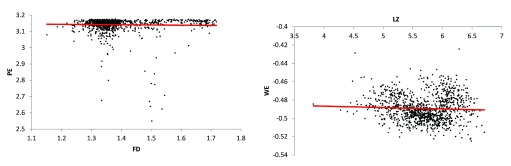
Scatter plots with linear trendlines for two pairs of insignificant, uncorrelated complexity measures. Two pairs of complexity measures of the EEG signals were insignificant and uncorrelated - PE & FD (r=-0.0255, p=0.3990) and WE & LZ (r=-0.0472, p=0.1174). There appears to be no non-linear components or any evidence of a clear relationship between these pairs of measures. *EEG = electroencephalogram; LZ = Lempel-Ziv algorithmic complexity; FD = fractal dimension estimate (Higuchi method); PE = permutation entropy; WE = Wiener entropy (also known as spectral flatness)*.

**Figure 3.**
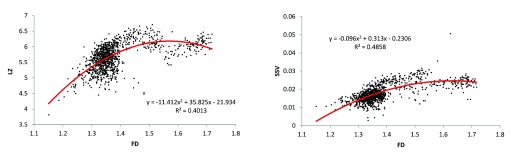
Scatter plots with binomial regression lines for potential non-linearly-related pairs of complexity measures. Two pairs of complexity measures of the EEG signals appeared to have noticeable non-linear relationships: (i) LZ and FD; and (ii) SSV and FD. Although these binomial relationships were - like their linear relationships - significant (p<0.0001), the binomial regressions produced less spread and appear to be truer representations of the relationships. *EEG = electroencephalogram; LZ = Lempel-Ziv algorithmic complexity; FD = fractal dimension estimate (Higuchi method); SSV = spectral structure variability*.

These relationships were visualised using scatter plots ( Figure 1 and Figure 2) to help determine if any of these relationships may be non-linear. Two such relationships - (i) LZ and FD, (ii) SSV and FD - appeared to follow a binomial trend ( Figure 3), and binomial regression improved these relationships greatly.

Calculated complexity measures for 1100 EEG recordings The following data are the results from MATLAB functions which calculated complexity measures for each EEG recording. The following data are the results from MATLAB functions which calculated complexity measures for each EEG recording. *ID = identification code as per Begleiter (1996); LZ = Lempel-Ziv algorithmic complexity; FD = fractal dimension estimate (Higuchi method); PE = permutation entropy; WE = Wiener entropy (also known as spectral flatness)* ( Burns & Rajan, 2015). Click here for additional data file. Copyright: © 2015 Burns T and Rajan R 2015 Data associated with the article are available under the terms of the Creative Commons Zero "No rights reserved" data waiver (CC0 1.0 Public domain dedication).

## Discussion and conclusions

Some - but not all - measures of complexity of EEG signals correlate, and to varying degrees of significance, e.g. we found no significant relationship between PE and FD but did find a significant relation between PE and LZ. To the best of our knowledge, this study represents the first report of such complexity measure differences in EEG signals. Of the many complexity measures available to researchers investigating EEG data, overreliance or overconfidence in any single measure therefore seems misplaced. As research groups who have attempted to classify or predict sleep stages or conscious states from EEG data have implicitly noted ( Susmáková & Krakovská, 2008; Sitt *et al.*, 2014; Weiss *et al.*, 2011), no individual measure can reliably predict all possibly relevant physiology. Instead, combinations of measures are needed. In the same way, no individual measurement of complexity can reliably predict all possibly relevant complexity.

In part, the results from this short study reflect on a more generalised ambiguity of the concept of ‘complexity’. Who is to say, after all, that more is revealed about ‘complexity’ by FD than LZ? It seems that it cannot be said that either elucidate more or less about ‘complexity’, since both ultimately treat it in a different way on even a conceptual basis. This further reiterates the primary finding of the present study: by multiplying measures we can reveal information which was previously hidden or unknown to us. However, there are two caveats to this: (1) not all information may be physiologically or otherwise relevant all of the time (or ever); and (2) different datasets may, due to their differences in nature, show different levels of covariance between complexity measures.

It would be interesting for future studies to analyse previously-noted complexity differences - e.g., between patients with and without Alzheimer's disease ( Dauwels *et al.*, 2010) - to determine if these differences were measuring the same difference. Our results suggests they may not be. And if this is the case, more might be gleaned from the available data if more measures were applied in combination. It could even be possible that there exists entirely separate complexity dimensions, along which patients progress at different rates. Such information could therefore contain even more physiological, clinical, or other significance than previously thought.

## Data availability

The data referenced by this article are under copyright with the following copyright statement: Copyright: © 2015 Burns T and Rajan R

Data associated with the article are available under the terms of the Creative Commons Zero "No rights reserved" data waiver (CC0 1.0 Public domain dedication). 

A copy of MATLAB functions used in this study has been uploaded to GitHub and can be accessed here: https://github.com/tfburns/MATLAB-functions-for-complexity-measures-of-one-dimensional-signals.

Results from these functions for the EEG data used can be found in the F1000Research repository (see below).

*F1000Research*: Dataset 1. Calculated complexity measures for 1100 EEG recordings. The data are the results from MATLAB functions which calculated complexity measures for each EEG recording, 10.5256/f1000research.6590.d48983 ( Burns & Rajan, 2015).
