# Supplementary material for: Combining complexity measures of EEG data: multiplying measures reveal previously hidden information
Source: F1000Res. 2015 Jun 1;4:137. [Version 1] doi: 10.12688/f1000research.6590.1 (PMC4648221; doi:10.12688/f1000research.6590.1)
Supplement: Peer review history

# Peer review: Referee response for version 1

**DOI:** 10.5256/f1000research.7077.r8846

**Published:** 2015-09-24

**Authors:** Alison Pack

**Affiliations:** 1 Neurological Institute, Columbia University, New York, NY, 10032, USA

This is a well written report addressing the utility of multiple measures used to evaluate EEG. EEG continues to be a valuable tool for research and clinical work. This paper nicely assesses through statistical analysis whether unique features of EEG can be helpful. The methods and analysis are well formulated. The conclusions are clearly articulated and are supported by research findings.

I have read this submission. I believe that I have an appropriate level of expertise to confirm that it is of an acceptable scientific standard.

# Peer review: Referee response for version 1

**DOI:** 10.5256/f1000research.7077.r8965

**Published:** 2015-06-25

**Authors:** Shifu Xiao

**Affiliations:** 1 Alzheimer’s Diagnosis and Treatment Centre, Shanghai Jiaotong University School of Medicine, Shanghai, 200030, China

This is an interesting article. The authors computed five complexity measures which were broadly representative, aiming to determine the correlations and differences between measures of complexity used in EEG signals study. Just as the findings of this study, more information can be revealed by multiplying measures instead of single complexity measures. Furthermore, the methods of combinations of complexity measures can be applied in future studies such as Alzheimer’s disease. With the coming of big data era, we need to adopt effective means to mine information hidden in many diseases. Perhaps, this paper gives us some reference.

I have read this submission. I believe that I have an appropriate level of expertise to confirm that it is of an acceptable scientific standard.
